# Birch Rust Spore Exposure and Upper Airway Symptom Dynamics in a Subarctic Region: Indications for a Novel Seasonal Allergen

**DOI:** 10.1002/clt2.70196

**Published:** 2026-08-09

**Authors:** Randi Falnes Olsen, Kristian Svendsen, Thorsten Graf, Annette Kuehn, Viera Stubnova, Martin Sørensen

**Affiliations:** ^1^ Regional Center for Asthma, Allergy and Intolerance University Hospital of North Norway Tromsø Norway; ^2^ Department of Clinical Medicine, Faculty of Health Sciences UiT – The Arctic University of Norway Tromsø Norway; ^3^ Department of Pharmacy UiT – The Arctic University of Norway Tromsø Norway; ^4^ Department of Infection and Immunity Luxembourg Institute of Health Luxembourg Luxembourg; ^5^ Finnmark Hospital Trust Kirkenes Norway

**Keywords:** airway allergy, birch rust fungi, seasonal allergy

## Abstract

**Background:**

Airway allergies affect 20%–30% of the global population, with pollen and fungal spores representing major environmental triggers. Although fungal spores are far more abundant than pollen, their allergenic role—particularly from phytopathogenic rust fungi such as birch rust (BR)—remains poorly understood. In Northern Norway, where seasonal dispersal of BR spores occurs with minimal overlap from other aeroallergen exposures, we investigated their potential contribution to autumnal airway symptoms by linking spore distribution with clinical outcomes.

**Methods:**

In this prospective study, 160 patients and 94 controls were followed during BR season (Aug‐Oct) and out of season (Jan‐Feb). Participants reported allergic rhinitis, conjunctivitis, and/or asthma. Symptom scores (Visual Analogue Scale), nasal airflow (Peak Nasal Inspiratory Flow), and lung function (spirometry) were recorded, alongside daily BR spore counts from three monitoring sites.

**Results:**

During BR season, patients showed significantly higher symptom severity (VAS difference 1.58, *p* < 0.01) and lower PNIF values (−12.2 L/min, *p* = 0.01) versus controls. Seasonal increases in symptoms were observed (1.70, *p* < 0.001), and anti‐allergic medication use was more frequent in patients (62.5% of patients vs. 1.1% of controls (*p* < 0.001). Symptom worsening was associated with reported proximity to birch forests (regression coefficient 0.46, *p* < 0.01, *R* ^2^ = 0.48).

**Conclusions:**

BR spore exposure was associated with increased autumnal airway symptoms, suggesting that BR may represent an underrecognized trigger. These findings highlight the need for further research, including allergen‐specific testing and dispersal modelling, to clarify their clinical relevance.

**Trial Registration:**

This study is registered at ClinicalTrials.gov (identifier: NCT05661812)

**Abbreviations**
BR: birch rust
FEV1: forced expiratory volume in one second
FVC: forced vital capacity
MeDALL questionnaire: Mechanisms of the Development of ALLergic diseases
PEF: peak expiratory flow
PNIF: peak nasal inspiratory flow
SD: standard deviation
VAS: visual analogue scale

## 1 Introduction

Airway allergies, primarily triggered by pollen and fungal spores, affect 20%–30% of the global population [1, 2, 3, 4, 5]. They markedly reduce quality of life, increase healthcare utilization, and impair productivity, accounting for over 20 million disability‐adjusted life years (DALYs) lost globally and annual healthcare costs exceeding 20 billion euros in Europe alone [4, 6, 7, 8]. Prevalence continues to rise across all age groups, driven by lifestyle and environmental changes—particularly air pollution and climate change— that reshape the human exposome, impair immune tolerance, and alter the diversity, distribution, and allergenicity of aeroallergens [2, 9, 10, 11, 12, 13].

Alongside well‐established pollen allergens, fungal spore exposure is increasingly recognized as a dynamic and clinically relevant component of the aeroallergen environment [14, 15, 16]. While long‐term trends in total annual spore concentrations vary geographically and may not consistently show overall increases, geographically heterogeneous shifts in seasonality, including earlier seasonal onset and prolonged exposure periods, have been reported [14, 17]. Atmospheric fungal spore concentrations often exceed pollen levels by 100–1000 times, and fungal spores are increasingly implicated as triggers of asthma exacerbations, as well as IgE‐mediated rhinitis and conjunctivitis [14, 15, 16, 18, 19, 20, 21, 22]. Despite an estimated 1–1.5 million fungal species worldwide, the WHO/IUIS allergen database lists only 113 aeroallergens from 30 species, and global sensitization to fungi is reported in only about 5% of the population [16, 23]. Most fungi therefore remain uncharacterized with respect to allergenicity and dispersal pattern. Given the species‐ and region‐specific nature of fungal allergy—and the accelerating influence of climate change—field studies in diverse environments are essential to identify novel aeroallergens, refine local exposure profiles, and guide prevention strategies [14, 23, 24].

Rust fungi (order *Puccinales*, phylum *Basidiomycota*) merit particular attention. These phytopathogenic fungi are widespread and exhibit intensified seasonal sporulation in response to favorable climatic fluctuations [14, 16]. Their airborne microspores (15–35 μm) are produced in vast quantities, readily inhaled, and their cell walls contain chitin —a known pro‐allergenic component [16, 25]. Despite this biological plausibility, medical evidence linking rust spores to allergic disease is limited, with only a few older studies suggesting such associations [15, 26].

*Melampsoridium betulinum*, the causal agent of birch rust (BR), is a rust fungus affecting birch trees. It is most common in pure birch forest stands within cool temperate and boreal zones of the Northern Hemisphere, particularly at higher altitudes. Infected leaves develop yellow pustules that release BR spores (urediniospores) during autumn. Unlike molds such as Cladosporium and Alternaria, which release spores in episodic bursts triggered by specific weather events, BR spores exhibit a distinct seasonal dispersal pattern characterized by day‐to‐day release over several weeks during their active season [27, 28]. Experimental work suggests that climatic conditions associated with warming temperatures may enhance BR proliferation and survival [27]. Consistent with this, regional monitoring data from Tromsø and Kirkenes indicate increasing BR spore concentrations in Northern Norway. Using multi‐year averages based on two equal five‐year periods, mean annual BR spore levels increased from approximately 1100 spores/m^3^ in 2010–2014 to approximately 2700 spores/m^3^ in 2020–2024 [28].

**FIGURE 1:**
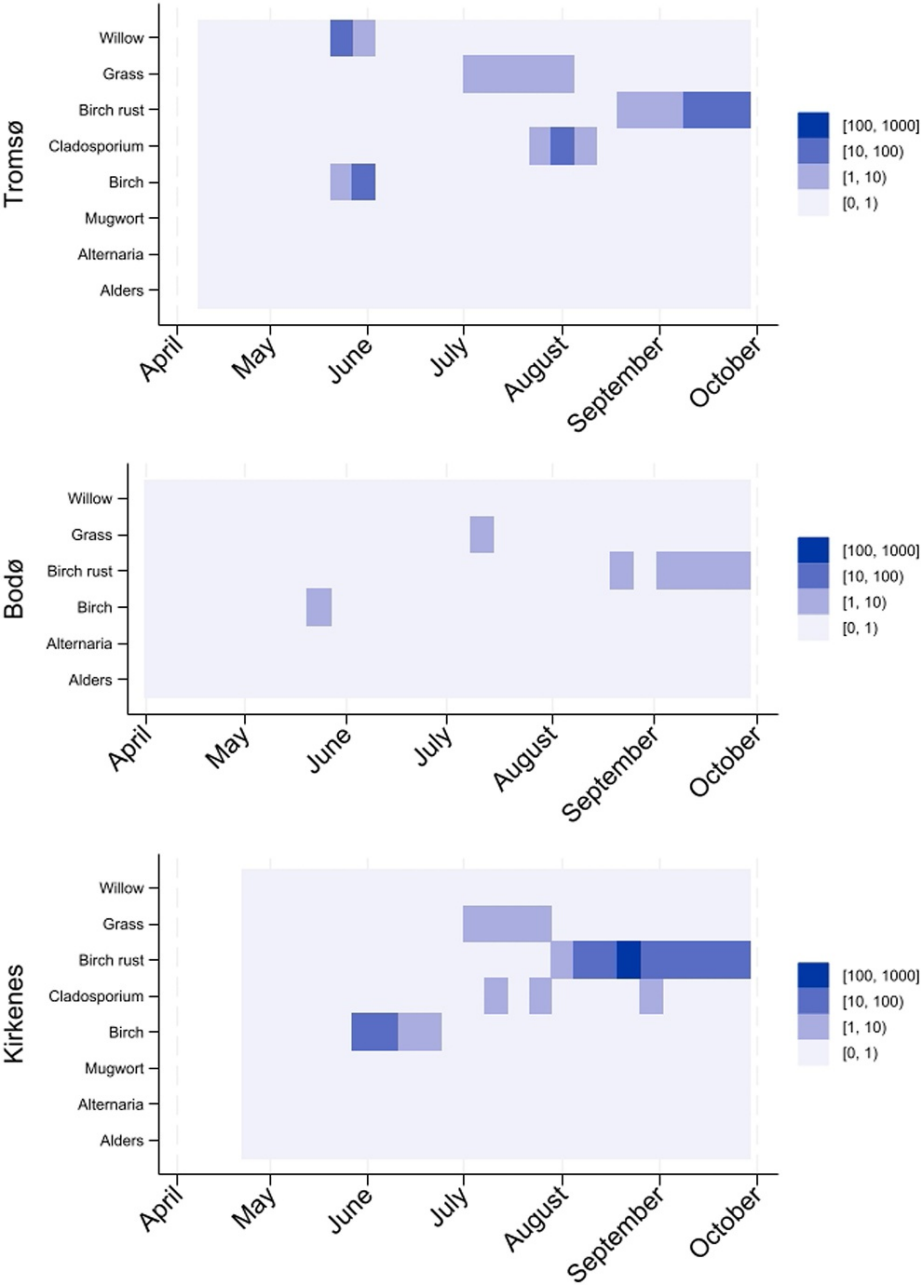
Distribution of allergenic pollen and spores in Tromsø, Bodø and Kirkenes during 2022, shown as mean weekly counts of pollen from trees (willow, birch, alders), grass, and mugwort, as well as spores from Cladosporium, Alternaria, and birch rust (Melampsoridium betulinum). Data are derived from the 2022 national aeroallergen monitoring program in Norway, provided by The National Pollen Forecast.

Clinical observations and preliminary data from Northern Norway (Tro‐BRA study) indicate a temporal association between BR spore dispersal and autumnal allergic airway symptoms, with prior investigations demonstrating both seasonal symptom patterns during the BR spore period and immunological sensitization to BR spores in susceptible individuals [29]. Patients frequently report recurrent episodes of rhino‐conjunctivitis and asthma exacerbations in late August and September— a period in which routine aerobiological monitoring in Northern Norway demonstrates minimal or absent dispersion of common seasonal aeroallergens (Figure 1). Birch and grass pollens peak earlier (May to mid‐August); *Cladosporium* spores, which coincide with grass pollen, rarely exceed clinical thresholds (3000 spores/m^3^); mugwort pollen and *Alternaria* spores are virtually absent; and long‐range transported ragweed (*Ambrosia*) pollen has not been detected. By contrast, BR spores dominate the aeroallergen profile from mid‐August to early October, with concentrations consistently surpassing *Cladosporium* over the past 15 years (e.g., 1487 vs. 59 spores/m^3^ in 2022) [28, 30].

Despite their prevalence and seasonal dominance during autumn, the potential of BR spores as aeroallergens has remained largely unexplored. In the context of ongoing climate change, seasonal co‐occurrence of pollen and fungal spores is becoming more frequent, increasing the cumulative exposure burden and potentially amplifying symptoms in susceptible individuals [31], thereby complicating the identification of specific etiological triggers. In addition, high airborne fungal spore concentrations may exert non‐specific irritative or toxic effects independent of allergic sensitization. Human challenge and epidemiological studies have suggested consistent lowest observed effect levels (LOELs) of approximately 10^5^ spores/m^3^ for diverse fungal species in non‐sensitized populations, indicating that high exposure alone may contribute to respiratory symptoms [32]. However, the near absence of competing seasonal aeroallergens from mid‐August to early October, together with the relatively stable dispersal and moderate concentrations of BR spores—remaining below levels typically associated with non‐allergic irritative effects—makes Northern Norway a unique natural setting in which to investigate the clinical relevance of BR exposure [28].

In this field study, we systematically examined whether exposure to BR spores is associated with airway allergy symptoms during autumn. By comparing allergic and non‐allergic individuals, we characterized symptom profiles, assessed severity, and mapped seasonal patterns against environmental BR data from three monitoring stations. This approach allowed us to investigate whether BR spore exposure is temporally associated with seasonal airway symptoms in a real‐world subarctic environment. Our aim was to evaluate the clinical relevance of BR spore exposure and thereby contribute to the identification of new, climate‐sensitive, region‐specific aeroallergens.

## 2 Material and Methods

### 2.1 Study Design and Study Population

**FIGURE 2:**
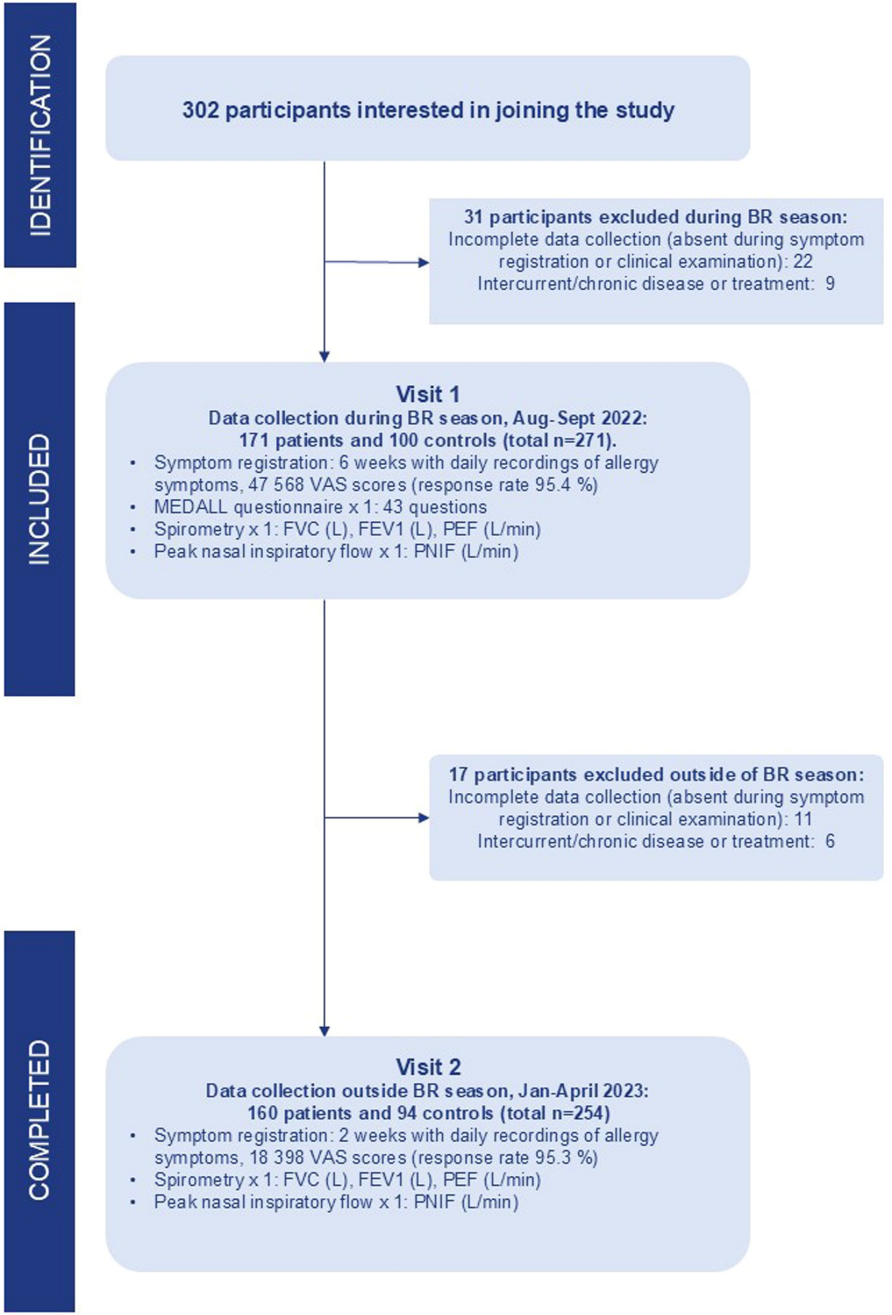
Flow chart of study design and data collection, showing participant recruitment, distribution into patient and control groups, and seasonal assessments (BR season and off‐season). Data sources included symptom registration (VAS), MeDALL questionnaire, lung function (spirometry), and Peak Nasal Inspiratory Flow (PNIF). FEV1: Forced Expiratory Volume in one second; FVC: Forced Vital Capacity; PEF: Peak Expiratory Flow; PNIF: Peak Nasal Inspiratory Flow; VAS: Visual Analogue Scale.

This prospective study included a final cohort of 254 adults from Northern Norway (Figure 2). A sample size calculation, based on a 2:1 patient‐to‐control ratio, indicated that a minimum of 217 participants was required. Eligible participants were aged 18–75 years and were recruited from five regions, affiliated with clinics in Kirkenes (11.0% of the participants), Alta (10.2% of the participants), Tromsø (47.6% of the participants), Bodø (11.8% of the participants) and Mo i Rana (19.3% of the participants). Participants were geographically distributed across large catchment areas and could reside considerable distances from their affiliated clinic, reflecting the dispersed population structure of Northern Norway. Clinic affiliation therefore represented recruitment and follow‐up centers rather than participants' residential locations. Recruitment strategies included posters, presentations on various platforms, and media outreach.

Participants were recruited into two groups: 160 patients and 94 controls. The patient group included individuals who reported allergic symptoms such as rhinitis, conjunctivitis, and/or asthma from August to October. Patients were excluded if they had perennial airway allergies (e.g., allergies to house dust mite (HDM) and/or pets) without exacerbations in autumn, conditions that could confound or mimic airway allergy symptoms, or were undergoing immunosuppressive drug treatment. The control group comprised individuals without allergic airway symptoms during fall or any known history of atopic diseases. This group was specifically recruited to ensure a clear clinical distinction from the allergic patient group, thereby reducing the risk of classifying patients as controls, or vice versa.

Data collection started in August 2022 and ended in April 2023, encompassing symptom records, questionnaires, and physical examinations using PNIF (Peak Nasal Inspiratory Flow) and spirometry (Figure 2). Assessments were conducted over two distinct periods: August‐September 2022 (during the BR spore season) and January to April 2023 (outside the BR spore season and during a period with minimal environmental aeroallergen exposure). Each participant recorded symptoms from August 15 to September 30, 2022, and from January 15 to February 3, 2023, while PNIF and spirometry measurements were performed once during each period. Due to logistical constraints, physical examinations outside the BR season were conducted after symptom recording but prior to the onset of the birch pollen season.

### 2.2 Symptom Registration and Questionnaire

A detailed participant survey was employed to collect data on symptoms indicative of BR allergy, current use of allergy and/or asthma medications, and on BR exposure (Supporting Information S1). Participants received emails every 2 days for 6 weeks during and for 2 weeks outside the BR spore season, asking them to rate both the current and the previous day using a Visual Analogue Scale (VAS).

Allergy symptoms were rated from 0 (no symptoms) to 10 (extremely pronounced symptoms). Participants provided separate symptom scores for conjunctivitis (eye symptoms), rhinitis (nasal symptoms), and asthma (coughing and dyspnea), as well as an overall score for total allergy symptoms (total symptoms).

Using the same VAS scale, participants also reported their same‐day proximity to birch forest environments. Proximity was defined as having been near or having spent time in an area perceived by the participant as birch forest during that day. No predefined distance or spatial criteria were provided; responses therefore reflected participants' subjective assessment of environmental contact. Ratings ranged from 0 (“not at all”) to 10 (“to a very large extent”). This measure was used as a subjective indicator of individual, same‐day BR exposure conditions, complementing environmental aeroallergen measurements.

Current use of allergy and asthma medications was reported as “yes” or “no,” with details on type. For analysis, medication use was defined as treatment on ≥ 6 days during the birch rust spore season and ≥ 2 days outside the season (≈ 15–16% of days). These cutoffs, based on clinical judgment and GINA guidelines (which define well‐controlled asthma as requiring reliever medication ≤ 2 times per week) [33], were chosen to capture treatment indicating insufficient symptom control rather than sporadic use for incidental symptoms.

VAS was employed to assess allergy symptoms, as it is a validated tool for capturing day‐to‐day variability. The VAS is regarded reliable, simple and intuitive, and does not require specific training. Symptom intensity was interpreted using the MACVIA‐ARIA–defined cut‐off values for allergic rhinitis: VAS > 5 indicating uncontrolled symptoms, 2–5 indicating partly controlled symptoms, and < 2 indicating controlled symptoms [34, 35, 36, 37].

To gather supplementary data on previous and current asthma, allergic rhinitis and eczema, we used a condensed version of the MeDALL questionnaire (Mechanisms of the Development of ALLergic diseases; EU project EU FP7‐CP‐IP) [38]. This version included 43 of the 88 questions from the original pediatric questionnaire (Supporting Information S1). The questionnaire was completed once during the autumn of 2022, and the participants completed it digitally from home.

Data collection from symptom registration and questionnaires was facilitated using Research Electronic Data Capture (REDCap).

### 2.3 Peak Nasal Inspiratory Flow and Spirometry

Peak nasal inspiratory flow (PNIF) and spirometry were conducted to evaluate obstruction in the upper and lower airways. These measurements were performed by trained assistants both during and outside the birch pollen season. Participant status was not blinded to the assistants.

PNIF and lung function were measured with patients seated, using an In‐check Inspiratory Flow Meter and a Micro 1 Diagnostic Spirometer. Standard instructions were provided in accordance with current guidelines, and the highest‐quality test results from three approved performances were used for analysis [39, 40, 41]. PNIF values > 120 L/min indicated absence of significant nasal obstruction [42, 43]. Forced vital capacity (FVC [L]), forced expiratory volume in one second (FEV1 [L]), and peak expiratory flow (PEF [L/min]) were recorded to assess lung function, with reference values from the European Community for Coal and Steel (ECCS) applied [44].

### 2.4 Outdoor Aeroallergen Monitoring

Airborne pollen and fungal spore data were obtained from the Norwegian National Pollen Forecast, using monitoring stations in Bodø, Tromsø, and Kirkenes [28]. Standardized sampling and analytical procedures, as routinely applied in the Norwegian monitoring program, are detailed in Supporting Information S1.

These data were used to describe the seasonal distribution and temporal dynamics of relevant aeroallergens during the study period (Figure 1).

Participants were geographically dispersed and often lived considerable distances from the monitoring station assigned to their region. Consequently, exposure estimates reflect area‐level aeroallergen conditions rather than individual point measurements.

### 2.5 Statistical Analyses

Statistical analyses were conducted using StataMP 17. Descriptive statistics were used to summarize participant characteristics and the prevalence of allergies, asthma, rhinitis, and eczema. For univariate comparisons of continuous variables, Student's *t*‐test was applied, while categorical variables in the MeDALL dataset and symptom registration data were compared using the Chi‐square test. VAS‐scored allergy symptoms were categorized, for example, as controlled (VAS < 2) or not controlled (VAS > 2).

Univariable and multivariable linear regression models were used to assess seasonal variations in average symptom severity and PNIF in relation to participant status, adjusting for relevant covariates (proximity to birch forest; anti‐allergic and anti‐asthmatic treatment; rhinitis in the last 12 months; asthma in the last 12 months; pollen allergy, perennial allergy; family history of atopy; and ever atopic disease). Various models, incorporating different sets of confounders, were evaluated using goodness‐of‐fit statistics, both during and outside of the birch rust season. Final model selection was based on statistical criteria (*R* ^2^, AIC, and BIC) and biological plausibility.

Symptom VAS scores were analyzed as continuous outcomes. Although bounded (0–10) and mildly skewed, particularly among controls, linear regression models were considered appropriate given their widespread use for patient‐reported scales and demonstrated robustness to moderate deviations from normality in epidemiological research [45, 46].

To adjust for multiple comparisons, the Benjamini–Hochberg procedure was applied to descriptive comparisons between patients and controls. Statistical significance was defined as *p* < 0.05 for all analyses.

### 2.6 Drop‐Out Analysis

Participants who did not complete the study were compared to the final study population based on data from the MeDALL questionnaire, PNIF measurements, spirometry results and in BR season symptom registration.

## 3 Results

### 3.1 Different Allergic Prevalence in the Two Study Groups

In this cohort, 160 patients with allergic airway symptoms during autumn were compared with 94 non‐allergic controls. As expected, the groups differed in atopic background. Patients had a markedly higher prevalence of rhinitis, asthma, eczema, and seasonal/perennial allergies compared with minimally atopic controls (all *p* < 0.001; Table 1).

**TABLE 1:** Demographic and clinical characteristics of the study population, including general and birch rust (BR) specific variables.

	Patients (*N* = 160)	Controls (*N* = 94)	*p*‐value
General			
Age (years)	47.4 (SD 12.4)	46.7 (SD 10.8)	0.53^†^
Female gender	122 (76.3%)	76 (80.9%)	0.39^‡^
Allergy background (MeDALL)			
Pollen allergy			
Birch	93 (58.1%)	3 (3.2%)	< 0.001^‡^
Grass	78 (48.8%)	1 (1.1%)	< 0.001^‡^
Mugwort	17 (10.6%)	1 (1.1%)	0.004^‡^
Total	108 (67.5%)	3 (3.2%)	< 0.001^‡^
Perennial allergy (house dust mite, pets)	95 (59.6%)	2 (2.1%)	< 0.001^‡^
Family history of atopy^a^	139 (86.9%)	52 (55.3%)	< 0.001^‡^
Ever atopic disease^b^	143 (89.4%)	19 (20.2%)	< 0.001^‡^
Current asthma^c^	73 (45.6%)	2 (2.1%)	< 0.001^‡^
Current rhinitis^c^	106 (66.3%)	0 (0%)	< 0.001^‡^
Current eczema^c^	48 (30.0%)	6 (6.4%)	< 0.001^‡^
Medical treatment (symptom registration)			
Anti‐allergic treatment^c^			
In BR season^c^	100 (62.5%)	1 (1.1%)	< 0.001^‡^
Outside BR season^c^	63 (39.6%)^e^	2 (2.1%)	< 0.001^‡^
Anti‐asthmatic treatment^c^			
In BR season^c^	53 (33.1%)	0	< 0.001^‡^
Outside BR season^c^	42 (26.4%)^e^	0	< 0.001^‡^
Birch rust exposure (symptom registration)			
Proximity to birch forest (mean VAS)^c^ ^,^ ^d^			
In BR season	3.09 (SD 2.04)	2.22 (SD 1.86)	< 0.001^†^
Outside BR season	1.26 (SD 2.08)^e^	1.14 (SD 1.74)	0.64^†^

*Note:* Data from the MeDALL questionnaire and symptom registration questionnaire. Statistical tests: ^†^ Student's t test; ^‡^ Chi‐square test.

Abbreviations: BR, birch rust; SD, standard deviation; VAS, visual analogue scale.

^a^ Parent, siblings, children, or grandchildren who have or have had asthma, eczema, airway allergies and/or food allergies.

^b^ Diagnosed by a physician and/or experienced as a child/adolescent with asthma, eczema, airway allergies and/or food allergies.

^c^ For more detailed definition, please refer to Supporting Information S1.

^d^ Self‐reported registration of having been near or spent time in a birch forest.

^e^ *N* = 159 due to missing symptom registration outside the BR season for one patient.

### 3.2 Increased Allergy Symptoms During BR Season

During BR season, patients exhibited significantly more severe allergic symptoms than controls, as reflected by total VAS scores (adjusted difference 1.58; *p* < 0.01). This finding was consistent across individual assessments of nasal, ocular and lower airway symptoms (Table 2). In unadjusted analysis, the difference in symptom severity was even more pronounced. Patients had a mean VAS score of 2.86 (SD 1.66, range 9.15), while controls had a mean score of 0.10 (SD 0.30, range 1.73), resulting in a VAS score difference of 2.76 (*p* < 0.01) (Supporting Information S1). Notably, 65% (*n* = 104) of the patients reported VAS scores > 2, compared with none of the controls.

**TABLE 2:** Mean difference in symptom severity and airway function between patients and controls, stratified by BR and non‐BR seasons.

Difference patients versus controls^a^	In BR spore dispersal season			Outside BR spore dispersal season^c^		
	Model 0	Model 1	Model 2	Model 0	Model 1	Model 2
Total symptoms^b^ (VAS)	2.76, *p* < 0.001	2.46, *p* < 0.001	1.58, *p* < 0.001	1.05, *p* < 0.001	1.05, *p* < 0.001	0.40, *p* = 0.08
Nasal symptoms^b^ (VAS)	2.54, *p* < 0.001	2.24, *p* < 0.001	1.30, *p* < 0.001	1.04, *p* < 0.001	1.03, *p* < 0.001	0.28, *p* = 0.25
Eye symptoms^b^ (VAS)	1.88, *p* < 0.001	1.63, *p* < 0.001	0.85, *p* = 0.001	0.69, *p* < 0.001	0.68, *p* < 0.001	0.06, *p* = 0.78
Asthma symptoms^b^ (VAS)	1.55, *p* < 0.001	1.28, *p* < 0.001	0.58, *p* = 0.03	0.54, *p* < 0.001	0.52, *p* < 0.001	0.003, *p* = 1.0
PNIF (L/min)^b^	−12.17, *p* = 0.01	−11.33, *p* = 0.02		−6.07, *p* = 0.19	−6.14, *p* = 0,18	
FEV1 (L)^b^	−0.22, *p* = 0.03	−0.19, *p* = 0.06		−0.19, *p* = 0.05	−0.18, *p* = 0.06	
FVC (L)^b^	−0.24, *p* = 0.05	−0.23, *p* = 0.06		−0.21, *p* = 0.08	−0.20, *p* = 0.10	

*Note:* Linear regression models: Model 0, unadjusted; Model 1, adjusted for proximity to birch forest; Model 2, adjusted for proximity to birch forest, anti‐allergic and anti‐asthmatic treatment, rhinitis in the last 12 months, asthma in the last 12 months, pollen allergy, perennial allergy, family history of atopy, and ever atopic disease.

Abbreviations: BR, birch rust; L, Liter; L/min, Liter/minute; PNIF, peak nasal inspiratory flow; SD, standard deviation; VAS, visual analogue scale.

^a^ Controls used as reference group.

^b^ For background data and variable definitions, see Supporting Information S1.

^c^ *N* = 253 due to missing symptom registration outside the BR season for one patient.

Outside the BR season, no statistically significant between‐group difference was observed after adjustment (Table 2). However, patients reported a mean total symptom VAS of 1.12 (SD 1.46, range 7.50) outside BR season, with 18% (*n* = 28) having scores > 2. Controls maintained a low symptom burden, with a mean VAS of 0.06 (SD 0.24, range 1.67) (Supporting Information S1).

**FIGURE 3:**
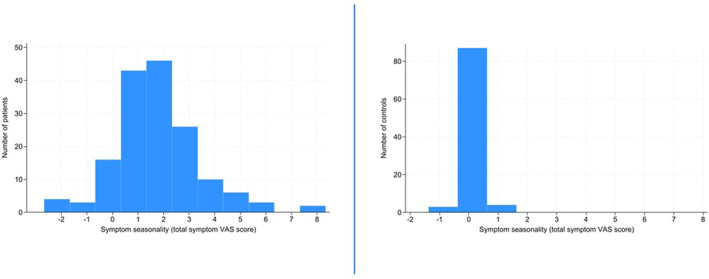
Symptom seasonality in patients and controls, measured as mean total symptom VAS scores. A value of 0 indicates no seasonal variation; VAS > 0 indicates more symptoms during the BR season than outside; VAS < 0 indicates more symptoms outside the BR season. A mean seasonal difference > 2 on the VAS was considered to reflect a clinically relevant worsening of allergic airway symptoms. BR: birch rust; VAS: Visual Analogue Scale.

A significant difference in symptom seasonality between patients and controls was observed, with patients showing a greater increase in VAS from outside to during BR season compared with controls (between‐group difference 1.70; *p* < 0.01; Figure 3). Forty percent (*n* = 63) of patients experienced a seasonal worsening > 2 VAS points, indicating a specific BR seasonal effect. In adjusted analyses, this seasonal between‐group difference was significant for total, nasal, and ocular symptoms, but not for asthma symptoms (Supporting Information S1).

### 3.3 Increased Medical Treatment During BR Season

Medication use rose among patients during the BR season. Anti‐allergic treatment increased significantly (40% vs. 26% off‐season; *p* < 0.01), while anti‐asthmatic treatment did not (22% vs. 21%; *p* = 0.19) (Table 1, Supporting Information S1). On average, patients used anti‐allergic medication for 19 days during the BR season, compared with 5 days outside. Controls used little or no medication year‐round.

### 3.4 Decreased PNIF During BR Season

During the BR season patients had lower PNIF than controls (mean difference 12.2 L/min, *p* = 0.01), and PNIF was lower in‐season than off‐season (difference 5.25 L/min, *p* = 0.01) (Table 2, Supporting Information S1). Mean PNIF in patients was 115.1 L/min (SD 35.7), indicating nasal obstruction during BR season, compared with 127.2 L/min (SD 35.4) in controls, which is within normal range.

In terms of lung function, FEV1 and FVC were consistently year‐round lower in patients than controls but showed no BR‐related seasonality (Table 2 and Supporting Information S1).

### 3.5 Allergic Symptoms Corresponded With Exposure to BR

**FIGURE 4:**
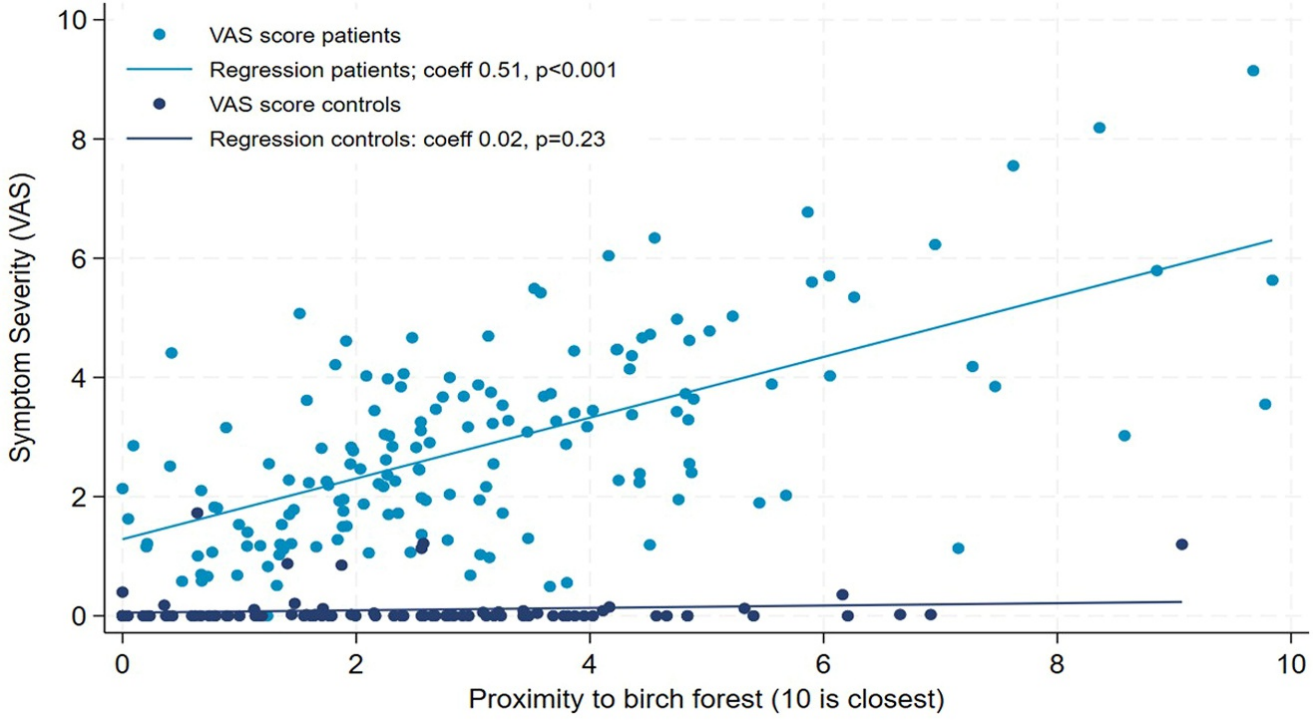
Correlation between symptom severity and proximity to birch forest during the BR season in patients and controls. Symptom severity was measured as mean total symptom VAS score, and proximity to birch forest was assessed using a VAS scale reflecting time spent in or near birch forest. BR: birch rust; VAS: Visual Analogue Scale.

A significant positive association between symptom severity and same‐day proximity to birch forest was observed among patients, but not in controls (Figure 4). This association remained significant after adjustment in linear regression, persisting among patients (regression coefficient 0.46, *p* < 0.01, *R* ^2^ = 0.48), but not among controls (regression coefficient 0.01, *p* = 0.53, *R* ^2^ = 0.12).

To account for geographical differences in exposure, analyses were stratified by study location. Seasonal BR spore counts varied across the three study locations, with annual totals of 105, 988, and 3370 spores/m^3^ air in Bodø, Tromsø, and Kirkenes, respectively [27]. Kirkenes consistently recorded the highest spore concentrations, corresponding to a greater reported symptom burden: the mean symptom VAS difference between patients and controls was 3.37 (*p* < 0.001), compared with 2.50 (*p* < 0.001) in Tromsø and 2.45 (*p* < 0.001) in Bodø (Supporting Information S2). In contrast, no significant association was found between Cladosporium spore counts and symptoms among the 28 patients in Kirkenes (Wilcoxon rank‐sum test).

### 3.6 No Differences Between Dropouts and Final Study Population

Baseline characteristics and in BR season symptom severity (mean total symptom VAS) did not differ between participants (*n* = 254) and dropouts (*n* = 17) (Supporting Information S3).

## 4 Summary of Key Findings

In summary, patients with allergic airway disease exhibited a clear seasonal increase in symptoms during the BR season, with greater symptom severity, increased use of anti‐allergic medication, and reduced PNIF compared with both controls and their off‐season measurements. By contrast, lung function parameters remained stable across seasons. Symptom fluctuations were temporally associated with indicators of BR exposure, including same‐day proximity to birch forest and regional spore counts.

## 5 Discussion

The rising prevalence of airway allergies and the influence of climate change on aeroallergen exposure highlight the importance of investigating novel allergenic sources [12]. To our knowledge, this is the first prospective cohort study to explore BR spores as potential contributors to seasonal allergic airway symptoms during autumn, building on prior immunological findings demonstrating sensitization to BR spores in susceptible individuals [29]. In a geographically defined setting, we observed distinct seasonal symptom patterns among patients, but not controls, occurring during the period of BR spore dispersal. While field studies are inherently subject to environmental variability and rely on participant‐reported outcomes, they provide real‐world insights that cannot be achieved in controlled settings [47]. By combining prospective symptom recordings, spore measurements from Northern Norway and participant‐reported same‐day exposure conditions, we demonstrated temporal associations between BR spore dispersal and airway symptoms, providing new insights into the potential role of BR spores as a region‐specific aeroallergen.

Our findings revealed significant differences in severity and seasonality of allergic airway symptoms between patients and controls, with a corresponding pattern in anti‐allergic medication use. Patients experienced notable seasonal fluctuations in symptom severity and treatment needs that coincided with the BR spore season, a pattern absent in non‐allergic controls. We acknowledge that inherent differences exist between patients and controls in terms of atopic status, allergic comorbidity, and polysensitization. This leads to differences in allergic susceptibility and an expected heightened reactivity among patients when exposed to both specific and non‐specific triggers, including irritants [15, 48, 49, 50]. Nevertheless, seasonal differences in airway symptoms persisted after adjustment for atopic covariates, supporting an association between the BR spore season and increased airway symptom burden among allergic individuals, consistent with a potential allergenic role of BR spores during autumn.

The observed symptom patterns cannot distinguish between IgE‐mediated responses, adjuvant effects related to polysensitization or co‐exposure, and non‐specific airway irritation. High concentrations of airborne particles may induce respiratory symptoms through irritative or inflammatory mechanisms independent of sensitization, and co‐exposure to other allergens may amplify symptoms in susceptible individuals [31, 32, 50]. However, in our setting, BR spores dominated the airborne allergen load, while measured concentrations remained below levels typically associated with irritative effects, thereby reducing potential confounding from both co‐exposure and high particulate environments. Although conclusions regarding allergenicity at the individual level remain limited, our findings demonstrate temporal symptom associations consistent with, but not proving, an allergic mechanism. Importantly, prior immunological findings from the Tro‐BRA study [29], demonstrating sensitization to BR spores in susceptible individuals, support the biological plausibility of BR spores acting as aeroallergens and provide context for the symptom associations observed in the present cohort.

Typical clinical manifestations of airway allergies, such as rhino‐conjunctivitis and asthma were specifically evaluated in the symptom registration. Rhinitis emerged as the most prominent allergic symptom during BR spore season, followed by conjunctivitis, whereas asthma symptoms were less pronounced. PNIF measurements confirmed seasonal nasal congestion among patients, supporting the reported rhinitis symptoms, while spirometry revealed no consistent changes in lung function. Although the observed reduction in PNIF was statistically significant, the magnitude of change was modest and should be interpreted primarily as an indicator of upper airway involvement rather than marked nasal obstruction.

In pollen‐induced seasonal allergies, rhino‐conjunctivitis typically dominates the clinical picture, whereas fungal spores, being smaller and more respirable, are more often associated with asthma exacerbations [15, 16, 21, 51, 52, 53, 54]. Our findings suggest that BR spore exposure predominantly affects the upper airways, thereby producing a symptom profile more like pollen allergy than classical fungal sensitization. However, the relatively mild asthma manifestations in our study may reflect the high prevalence of year‐round anti‐asthmatic treatment among patients, which could have attenuated bronchial hyper‐reactivity and reduced the risk of exacerbations. In addition, the use of a single spirometry measurement per participant in each season may not adequately capture the episodic nature of asthma, as sporadic testing is ill‐suited to identifying transient, exposure‐induced airway obstruction [2]. Taken together, our findings suggest a clinical profile in which upper airway symptoms dominate, while lower airway involvement remains uncertain and cannot be excluded.

To better understand the observed symptom profiles and seasonal variation, we examined associations with both individual exposure characteristics and regional BR spore concentrations. Patients reporting greater perceived proximity to birch forests experienced more severe airway symptoms, whereas no such association was observed among controls. Although this measure reflects self‐reported environmental contact rather than objectively quantified exposure, the exposure–symptom relationship was supported by parallel regional patterns, with stronger symptom severity observed in areas with higher BR spore concentrations, such as Kirkenes, compared with regions with lower levels, including Tromsø and Bodø. Interpretation of fungal allergenicity is often complicated by diverse airborne spore mixtures [14]. However, autumn aerobiology in Northern Norway is characterized by minimal dispersion of other clinically relevant aeroallergens, resulting in a comparatively homogeneous exposure environment dominated by BR spores [28]. This ecological specificity strengthens the observed exposure–symptom associations and supports a possible role of BR spores as a regionally relevant seasonal aeroallergen.

This study suggests that BR spores may represent an underrecognized contributor to seasonal allergic symptoms in autumn. To address our research objectives, we recruited allergic patients together with non‐allergic controls from the general population, creating a study cohort that allowed for clear contrasts between symptomatic individuals and unaffected comparators, thereby reducing the risk of misclassification. The longitudinal design, with assessments of clinical data both during and outside the BR season, enabled us to identify and compare symptoms in exposed and non‐exposed periods. This effectively illustrates a key indicator of allergy: the occurrence of symptoms during exposure and recovery during non‐exposure. Adjustment for potential confounders, including atopic constitution, coexisting allergies, rhinitis, and asthma, further strengthened the precision of our estimates. Environmental data on BR spore spread were obtained from actual counts at three locations, allowing comparison of regional spore levels with local symptom patterns while limiting potential environmental confounding. In addition, participants reported perceived proximity to birch forest environments as a subjective indicator of same‐day exposure conditions. Together, these complementary exposure indicators demonstrated a consistent symptom–exposure relationship and provided converging individual‐ and regional‐level support for the relevance of BR spores in autumn airway symptom expression.

Some limitations should be acknowledged. Self‐reported symptoms may introduce selection and recall bias, although regular symptom registration helps mitigate this risk. Tracking participants over just one BR season limits assessment of reproducibility across seasons—a key characteristic of allergic disease—and may not capture the full variability in BR levels. Individual BR spore exposure could not be measured directly, and daily activities, locations, and microenvironments may have influenced actual exposure, restricting our analysis to seasonality and temporal associations rather than precise individual symptom–exposure relationships. In addition, regional BR spore counts from monitoring stations provided an overall measure of aeroallergen levels, but data were unavailable from two study locations (Alta and Mo i Rana), limiting exposure–symptom stratification to three locations (Bodø, Tromsø, and Kirkenes). Similar feasibility challenges in exposure characterization have been reported in early investigations of emerging fungal aeroallergens such as *Alternaria*, where epidemiological associations preceded detailed mechanistic understanding [55, 56]. Accordingly, the present study focuses on temporally resolved population‐level associations as an initial step in evaluating birch rust as a candidate seasonal airway allergen. Future studies incorporating multi‐season monitoring, improved individual exposure assessment, and objective diagnostic measurements will be important to further clarify the clinical relevance of BR spore exposure.

From a clinical perspective, the findings of the present study indicate that clinicians should consider birch rust as a possible cause of seasonal airway allergy during autumn in northern Fennoscandia. Although commercial diagnostic tools for assessing sensitization are currently lacking, specific IgE reactivity to BR spores has previously been demonstrated [29], reinforcing the clinical relevance of these findings.

## 6 Conclusion

This study suggests that BR spores are a clinically relevant aeroallergen candidate associated with seasonal autumnal airway symptoms, potentially improving understanding of symptom patterns and opportunities for targeted treatment.

## Author Contributions

**Randi Falnes Olsen:** conceptualization, data curation, formal analysis, investigation, visualization, writing – original draft, writing – review and editing. **Kristian Svendsen:** data curation, formal analysis, validation, visualization, writing – review and editing. **Thorsten Graf:** writing – review and editing. **Annette Kuehn:** writing – review and editing, conceptualization. **Viera Stubnova:** writing – review and editing, conceptualization. **Martin Sørensen:** conceptualization, project administration, funding acquisition, supervision, writing – review and editing.

## Funding

This work was supported by Northern Norway Regional Health Authority, University Hospital of North Norway, The Norwegian Asthma and Allergy Association, research fund, Odd Berg Group, Medical research fund and Ministry of Higher Education and Research (MESR), Luxembourg.

## Ethics Statement

The study was conducted in accordance with Norwegian legislation and to the Declaration of Helsinki (General Assembly, Fortaleza, Brazil, 2013 and the subsequent updates). Ethical approval was obtained from The Regional Committee for Medical and Health Research Ethics (REK Norway; approval no. 272466).

## Consent

Participation was voluntary, and written informed consent was obtained from all participants.

## Conflicts of Interest

The authors declare no conflicts of interest.

## Supporting information


Supporting Information S1

Supporting Information S2

Supporting Information S3

## Data Availability

The data that support the findings of this study are available on request from the corresponding author. The data are not publicly available due to privacy or ethical restrictions.
