# Supplementary material for: Birch Rust Spore Exposure and Upper Airway Symptom Dynamics in a Subarctic Region: Indications for a Novel Seasonal Allergen
Source: Clin Transl Allergy. 2026 Aug 9;16(8):e70196. doi: 10.1002/clt2.70196 (PMC13454375; doi:10.1002/clt2.70196)
Supplement: Supplementary file 1 [file CLT2-16-e70196-s001.docx]

|  | | **Kirkenes (N=28)** | | | **Tromsø (N=121)** | | | **Bodø (N=30)** | | |
| --- | --- | --- | --- | --- | --- | --- | --- | --- | --- | --- |
| **ALLERGIC SYMPTOMS** | | Patients (N=19) | Controls (N=9) | P-value | Patients (N=76) | Controls (N=45) | P-value | Patients (N=12) | Controls (N=18) | P-value |
| **Total symptoms (mean VAS)^1^** | |  | | | | | | | | |
|  | In BR season^5^ | 3.58 (SD 1.81) | 0.21 (SD 0.57) | <0.001^†^ | 2.61 (SD 1.70) | 0.11 (SD 0.30) | <0.001^†^ | 2.45 (SD 1.36) | 0.02 (SD 0.05) | <0.001^†^ |
|  | Outside BR season6 | 1.32 (SD 1.50) | 0.02 (SD 0.05) | 0.02^†^ | 0.77 (SD 0.96)* | 0.10 (SD 0.33) | <0.001^†^ | 0.83 (SD 1.30) | 0.03 (SD 0.12) | 0.01^†^ |
| **Nasal symptoms (mean VAS)^2^** | |  | | | | | | | | |
|  | In BR season^5^ | 3.43 (SD 2.11) | 0.30 (SD 0.52) | <0.001^†^ | 2.49 (SD 1.72) | 0.20 (SD 0.35) | <0.001^†^ | 2.24 (SD 1.39) | 0.16 (SD 0.30) | <0.001^†^ |
|  | Outside BR season^6^ | 1.52 (SD 1.50) | 0.73 (SD 1.25) | 0.18^†^ | 0.92 (SD 0.99)* | 0.20 (SD 0.43) | <0.001^†^ | 0.62 (SD 0.91) | 0.18 (SD 0.32) | 0.07^†^ |
| **Eye symptoms (mean VAS)^3^** | |  | | | | | | | | |
|  | In BR season^5^ | 2.21 (SD 1.79) | 0.01 (SD 0.02) | 0.001^†^ | 1.69 (SD 1.73) | 0.02 (SD 0.05) | <0.001^†^ | 1.05 (SD 1.03) | 0.00 (SD 0.01) | <0.001^†^ |
|  | Outside BR season^6^ | 0.82 (SD 1.41) | 0 | 0.10^†^ | 0.47 (SD 0.80)* | 0.05 (SD 0.23) | 0.001^†^ | 0.35 (SD 0.61) | 0.07 (SD 0.21) | 0.09^†^ |
| **Asthma symptoms (mean VAS)^4^** | |  | | | | | | | | |
|  | In BR season^5^ | 1.99 (SD 2.16) | 0.24 (SD 0.69) | 0.03^†^ | 1.48 (SD 1.80) | 0.05 (SD 0.19) | <0.001^†^ | 1.17 (SD 1.45) | 0.04 (SD 0.12) | 0.003^†^ |
|  | Outside BR season^6^ | 1.02 (SD 1.70) | 0.62 (SD 1.23) | 0.54^†^ | 0.50 (SD 0.85)* | 0.04 (SD 0.15) | <0.001^†^ | 0.18 (SD 0.35) | 0.07 (SD 0.29) | 0.36^†^ |

**Supplementary 7: Mean VAS for allergic symptoms in patients and controls across Kirkenes, Tromsø, and Bodø.**

**Abbreviations:** BR, birch rust; SD, standard deviation; VAS, visual analogue scale.
**Statistical tests:** †Student`s *t* test.

^1^: Grading of overall allergic symptoms.

^2^: Grading of rhinitis symptoms: sneezing, itching, runny nose, nasal congestion, forehead pressure/pain, and throat mucus.

^3^: Grading of conjunctivitis symptoms: itching, redness, and tearing.

^4^: Grading of asthma symptoms: chest tightness, coughing, wheezing, and shortness of breath.

^5^: Total registration period of 47 days in the season of BR spore dispersal, from August 15 September 30, 2022.

^6^: Total registration period of 19 days outside the season of BR spore dispersal, from January 15 to February 3, 2023.

*N=75 due to missing symptom registration outside the BR season for one patient.
