# Supplementary material for: Birch Rust Spore Exposure and Upper Airway Symptom Dynamics in a Subarctic Region: Indications for a Novel Seasonal Allergen
Source: Clin Transl Allergy. 2026 Aug 9;16(8):e70196. doi: 10.1002/clt2.70196 (PMC13454375; doi:10.1002/clt2.70196)
Supplement: Supplementary file 2 [file CLT2-16-e70196-s002.docx]

**Supplementary 8:** Demographic and clinical characteristics of dropouts (N=17) compared to the study population (N=254).

Data from MeDALL questionnaire, PNIF/spirometry and in BR season symptom registration.

|  | | **Total dropouts**  **(N=17)** | **Total study population (N=254)** | **P-values (dropouts vs study population)** |
| --- | --- | --- | --- | --- |
| **GENERAL** | |  | | |
| **Patient percentage** | | 11 (64.7 %) | 160 (63.0 %) | 0.55^‡^ |
| **Age (years)** | | 40.5 (SD 11.3) | 47.1 (SD 11.9) | 0.03^†^ |
| **Female Gender** | | 12 (70.6 %) | 198 (78.0 %) | 0.55^‡^ |
| **ALLERGY BACKGROUND** | |  | | |
| **Pollen Allergy** | |  |  |  |
|  | Birch | 5 (29.4 %) | 96 (37.8 %) | 0.61^‡^ |
|  | Grass | 6 (35.3 %) | 79 (31.1 %) | 0.79^‡^ |
|  | Mugwort | 2 (11.8 %) | 18 (7.1 %) | 0.36^‡^ |
|  | Total | 6 (35.3 %) | 111 (43.7 %) | 0.62^‡^ |
| **Perennial Allergy (house dust mite, pets)** | | 4 (23.5 %) | 97 (38.2 %) | 0.30^‡^ |
| **Family history of Atopy^1^** | | 10 (58.8 %) | 191 (75.2 %) | 0.16^‡^ |
| **Ever Atopic Disease^2^** | | 7 (41.2 %) | 162 (63.8 %) | 0.07^‡^ |
| **Current Asthma^3^** | | 4 (23.5 %) | 75 (29.5 %) | 0.79^‡^ |
| **Current Rhinitis^4^** | | 6 (35.3 %) | 106 (41.7 %) | 0.80^‡^ |
| **Current Eczema^5^** | | 3 (17.6 %) | 54 (21.3 %) | 1.00^‡^ |
| **AIRWAY FUNCTION** | |  | | |
| **PNIF^6^ (L/min)** | | 104.1 (SD 32.4) | 119.6 (SD 36.0) | 0.09^†^ |
| **Spirometry** | |  |  |  |
|  | FEV1 (L) | 3.36 (SD 0.50) | 3.11 (SD 0.76) | 0.18^†^ |
|  | FVC (L) | 4.19 (SD 0.57) | 3.98 (SD 0.93) | 0.36^†^ |
|  | PEF (L/min) | 476.3 (SD 100.5) | 480.7 (SD 101.3) | 0.86^†^ |
| **SYMPTOM REGISTRATION** | |  |  |  |
| **Total symptoms (mean VAS)** | | 2.42 (SD 2.05) | 1.82 (SD 1.85) | 0.16^#^ |

**Abbreviations:** SD, standard deviation; VAS, visual analogue scale.
**Statistical tests:** †Student`s *t* test; ‡Chi-square test, #Wilcoxon rank sum test.

^1^: Parent, siblings, children, or grandchildren who have or have had asthma, eczema, airway allergies and/or food allergies.

^2^: Diagnosed by a physician and/or experienced as a child/adolescent with asthma, eczema, airway allergies and/or food allergies.

^3^: At least two of the following 3 criteria: 1) self-reported doctor-diagnosed asthma ever, 2) any indicative symptom in the last 12 months (wheezing, shortness of breath, dry cough at night), and 3) use of asthma medication in the past 12 months (1).

^4^: At least two of the following 3 criteria: 1) self-reported doctor-diagnosed allergic rhinitis ever, 2) symptoms of sneezing, a runny or blocked nose, and 3) itchy, red and watery eyes after exposure to furred pets, pollen or house dust mite [42].

^5^: At least two of the following 3 criteria: 1) self-reported doctor-diagnosed eczema ever, 2) dry skin, itchy rashes for two weeks or more in the past 12 months, and 3) self-reported eczema combined with use of topical corticosteroids in the past 12 months (2).

^6^: Peak Nasal Inspiratory Flow.

**References**

1.	Gough H, Grabenhenrich L, Reich A, Eckers N, Nitsche O, Schramm D, et al. Allergic multimorbidity of asthma, rhinitis and eczema over 20 years in the German birth cohort MAS. Pediatr Allergy Immunol. 2015;26(5):431–7.

2.	Ballardini N, Bergström A, Wahlgren C-F, van Hage M, Hallner E, Kull I, et al. IgE antibodies in relation to prevalence and multimorbidity of eczema, asthma, and rhinitis from birth to adolescence. Allergy. 2016;71(3):342–9.
