# Supplementary material for: Birch Rust Spore Exposure and Upper Airway Symptom Dynamics in a Subarctic Region: Indications for a Novel Seasonal Allergen
Source: Clin Transl Allergy. 2026 Aug 9;16(8):e70196. doi: 10.1002/clt2.70196 (PMC13454375; doi:10.1002/clt2.70196)
Supplement: Supplementary file 3 [file CLT2-16-e70196-s003.docx]

**SUPPLEMENTARY 1-5 AND SUPPLEMENTARY 7**

**Supplementary 1: English version of questionnaires for symptom registration and condensed version of MeDALL**

**Symptom Registration Nor-BRA**

To be sent via email for electronic completion every other day during two periods:

- August 15 to September 30, 2022
- January 15 to February 3, 2023

For questions 1-5 and 8-12, you are asked to rate the allergic symptoms you experienced yesterday and today on a scale from 0 to 10, where 0 indicates no symptoms and 10 indicates severely disruptive symptoms:

1. Overall, how bothersome were your allergy symptoms YESTERDAY?
2. How bothersome were your nasal symptoms (e.g., sneezing, itching, runny nose, nasal congestion, forehead pressure/pain, throat mucus) YESTERDAY?
3. How bothersome were your eye symptoms (e.g., itching, redness, and tearing) YESTERDAY?
4. How bothersome were your asthma symptoms (e.g., chest tightness, coughing, wheezing, and shortness of breath) YESTERDAY?
5. How bothersome were your skin symptoms (e.g., itching, dry skin, eczema) YESTERDAY?
6. To what extent did allergy symptoms affect your schoolwork, job, or leisure activities YESTERDAY? (0 = not at all, 10 = to a very large extent)
7. To what extent were you near or spent time in the birch forest YESTERDAY? (0 = not at all, 10 = to a very large extent)
8. Did you use any medication for your allergy YESTERDAY?
   Yes or No
9. Select the medications you used YESTERDAY:

Medications for nasal and/or eye symptoms:
• Eye drops such as:
o Livostin, Zaditen, Opatanol, Emadine, Spersallerg, Lomudal, Lecrolyn, Cleye, Tilavist = 1
• Cortisone eye drops such as:
o Spersadex, Softacort, Monopex, Ozurdex, Isopto-Maxidex, Ultracortenol, Triesence = 2
• Nasal decongestants such as:
o Rhinox, Otrivin, Dexyl, Zymelin, Otrivin Comp, Zycomb = 3
• Antihistamine nasal spray such as:
o Livostin = 4
• Cortisone nasal spray such as:
o Avamys, Nasonex, Flutide Nasal, Budesonide, Rhinocort, Fluticasone, Mometasone, Nasacort, Livicort, Otrason, Flurhin = 5
• Cortisone + antihistamine nasal spray such as:
o Dymista = 6
• Antihistamine tablets such as:
o Zyrtec, Cetirizine, Cetimax, Aerius, Telfast, Xyzal, Loratadine, Clarityn, Ebastine, Kestine, Zilas, Caredin, Polaramin, Phenergan, Alzyr, Atarax, Vallergan, Alimemazine = 7
• Cortisone tablets such as:
o Prednisolone, Cortisone, Dexamethasone, Medrol, Methylprednisolone, Betapred, Neofordex, Alkindi, Plenadren = 8
• Cortisone injections = 9

Medications for asthma or breathing difficulties:

• Short-acting bronchodilators such as:
o Bricanyl, Ventoline, Airomir, Buventol = 1

• Short-acting bronchodilators such as:
o Atrovent, Ipraxa, Spiriva, Eklira, Seebri, Incruse, Ultibro, Anora = 2

• Long-acting bronchodilators such as:
o Serevent, Oxis, Onbrez, Spiolto, Striverdi, Duaklir, Foradil = 3

• Anti-inflammatory medications such as:
o Flutide, Pulmicort, Aerobec, Alvesco, Beclomet, Giona, Asmanex, Becotide, Budesonide, Fluticasone = 4

• Long-acting bronchodilators + anti-inflammatory medications such as:
o Symbicort, Seretide, Flutiform, Inuxair, Serkep, Relvar, DuoResp, Airflusal, Bufomix, Salmex = 5

• Tablets such as:
o Singulair or Montelukast = 6

• Others such as:
o Trimbow, Trelegy = 7

10. Overall, how bothersome were your allergy symptoms TODAY?
11. How bothersome were your nasal symptoms (e.g., sneezing, itching, runny nose, nasal congestion, forehead pressure/pain, throat mucus) TODAY?
12. How bothersome were your eye symptoms (e.g., itching, redness, and tearing) TODAY?
13. How bothersome were your asthma symptoms (e.g., chest tightness, coughing, wheezing, and shortness of breath) TODAY?
14. How bothersome were your skin symptoms (e.g., itching, dry skin, eczema) TODAY?
15. To what extent did allergy symptoms affect your schoolwork, job, or leisure activities YESTERDAY? (0 = not at all, 10 = to a very large extent)
16. To what extent were you near or spent time in the birch forest TODAY? (0 = not at all, 10 = to a very large extent)
17. Did you use any medication for your allergy TODAY?
    Yes or No
18. Select the medications you used TODAY:

Medications for nasal and/or eye symptoms:
• Eye drops such as:
o Livostin, Zaditen, Opatanol, Emadine, Spersallerg, Lomudal, Lecrolyn, Cleye, Tilavist = 1
• Cortisone eye drops such as:
o Spersadex, Softacort, Monopex, Ozurdex, Isopto-Maxidex, Ultracortenol, Triesence = 2
• Nasal decongestants such as:
o Rhinox, Otrivin, Dexyl, Zymelin, Otrivin Comp, Zycomb = 3
• Antihistamine nasal spray such as:
o Livostin = 4
• Cortisone nasal spray such as:
o Avamys, Nasonex, Flutide Nasal, Budesonide, Rhinocort, Fluticasone, Mometasone, Nasacort, Livicort, Otrason, Flurhin = 5
• Cortisone + antihistamine nasal spray such as:
o Dymista = 6
• Antihistamine tablets such as:
o Zyrtec, Cetirizine, Cetimax, Aerius, Telfast, Xyzal, Loratadine, Clarityn, Ebastine, Kestine, Zilas, Caredin, Polaramin, Phenergan, Alzyr, Atarax, Vallergan, Alimemazine = 7
• Cortisone tablets such as:
o Prednisolone, Cortisone, Dexamethasone, Medrol, Methylprednisolone, Betapred, Neofordex, Alkindi, Plenadren = 8
• Cortisone injections = 9

Medications for asthma or breathing difficulties:

• Short-acting bronchodilators such as:
o Bricanyl, Ventoline, Airomir, Buventol = 1

• Short-acting bronchodilators such as:
o Atrovent, Ipraxa, Spiriva, Eklira, Seebri, Incruse, Ultibro, Anora = 2

• Long-acting bronchodilators such as:
o Serevent, Oxis, Onbrez, Spiolto, Striverdi, Duaklir, Foradil = 3

• Anti-inflammatory medications such as:
o Flutide, Pulmicort, Aerobec, Alvesco, Beclomet, Giona, Asmanex, Becotide, Budesonide, Fluticasone = 4

• Long-acting bronchodilators + anti-inflammatory medications such as:
o Symbicort, Seretide, Flutiform, Inuxair, Serkep, Relvar, DuoResp, Airflusal, Bufomix, Salmex = 5

• Tablets such as:
o Singulair or Montelukast = 6

• Others such as:
o Trimbow, Trelegy = 7

**MeDALL questionnaire Nor-BRA**

Asthma and dyspnoe

**1) In the past 12 months, have you had wheezing or whistling in your chest?**
If no: go to question 5
If yes:

**2) How many times have you experienced these symptoms in the past 12 months?**

- 1-3
- 4-12
- More than 12

**3) In the past 12 months, have you had trouble sleeping or woken up due to wheezing or whistling in your chest?**

- No
- Less than once a week
- 1 or more times a week

**4) In the past 12 months, have you missed school/work due to wheezing or whistling in your chest?**

- No
- Yes

**5) In the past 12 months, have you experienced breathing difficulties (shortness of breath, chest tightness, wheezing or whistling in your chest)?**

- No
- Yes

**6) In the past 12 months, have you had wheezing or whistling in your chest, shortness of breath, or troublesome coughing in connection with any of the following?**

- No, I have not had symptoms with any of these
- Cold air or fog
- Pets
- Birch pollen
- Grass pollen
- Mugwort pollen
- Air pollutants, tobacco smoke, strong odors, cooking fumes
- Between August 1 and November 1

**7) In the past 12 months, have you had wheezing or whistling in your chest, shortness of breath, or troublesome coughing in connection with physical exertion?**

- No
- Yes

**8) In the past 12 months, have you used any medications for asthma or breathing difficulties?**

- No, go to question 14
- Yes

**9) Which medications for asthma or breathing difficulties have you used in the past 12 months?**

- Bronchodilator: Bricanyl, Ventoline, Airomir, or Buventol
- Pulmicort, Flutide, Becotide, Giona, Beclomet, AeroBec, Budesonide, Alvesco, Fluticasone, or Asmanex
- Symbicort, Seretide, Oxis, Serevent, Onbrez, or Striverdi
- Seretide, Symbicort, Flutiform, Inuxair, Serkep, Relvar, DuoResp, Airflusal, Bufomix, or Salmex
- Atrovent, Ipraxa, Spiriva, Eklira, Seebri, Incruse, Duaklir, Ultibro, or Anora
- Trimbow or Trelegy
- Singulair or Montelukast tablets

**10) If you use bronchodilator medication (Bricanyl, Ventoline, Airomir, or Buventol), how often do you use it in a typical week?**

- Less than 2 times per week
- 2 times or more per week

**11) In the past 12 months, have you taken corticosteroid tablets for asthma or breathing difficulties?**

- No
- Yes

**12) In the past 12 months, have you had a dry cough at night without having a cold at the same time?**

- No
- Yes

**13) Have you been troubled by coughing most of the time, for at least 3 months per year?**

- No
- Yes. > How many years?

**14) Have you been troubled by mucus from your chest most of the time, for at least 3 months per year?**

- No
- Yes. > How many years?

**15) If you have breathing difficulties or asthma, has it:**

- Limited you in schoolwork/work?
- Limited you in leisure activities?
- Limited you from being outdoors?

**Skin Issues and Eczema**

**16) In the past 12 months, have you had problems with dry skin?**

- No -> go to question 18
- Yes

**17) In the past 12 months, have you applied moisturizing cream/lotion because of dry skin?**

- No
- Yes, less than 1 month
- Yes, 1-6 months
- Yes, more than 6 months

**18) In the past 12 months, have you had itchy rashes?**

- No > go to question 23
- Yes

**19) How long do your itchy rashes usually last?**

- Less than 1 week
- 1-2 weeks
- More than 2 weeks

**20) In which period(s) of the year have you had itchy rashes in the past 12 months? (You may select multiple options)**

- January - July
- February - August
- March - September
- April - October
- May - November
- June - December

**21) Has the itchy rash disappeared completely at any point in the past 12 months?**

- No
- Yes

**22) In the past 12 months, have you had difficulty sleeping or woken up due to itchy rashes?**

- No
- Less than once a week
- 1 or more times a week

**23) In the past 12 months, have you had eczema?**

- No > go to question 27
- Yes

**24) How long have you had eczema in total during the past 12 months?**

- Less than 1 month
- 1-3 months
- 3-6 months

**25) In the past 12 months, have you applied corticosteroid cream because of eczema?**

- No
- Yes, less than 1 month
- Yes, 1-6 months
- Yes, more than 6 months

**26) If you have skin issues or eczema, has it:**

- Limited you in schoolwork/work?
- Limited you in leisure activities?
- Limited you from being outdoors?

**Nose or Eye Symptoms**

**27) In the past 12 months, have you had sneezing, itchy nose, runny nose, or nasal congestion for more than 4 days without having a cold at the same time?**

- No, go to question 30
- Yes

**28) How long have you had these symptoms without having a cold at the same time in the past 12 months?**

- Less than 1 month
- 1-3 months
- 3-6 months
- More than 6 months

**29) In the past 12 months, have these nasal symptoms occurred at the same time as itchy, watery eyes?**

- No
- Yes

**30) During which period(s) have you experienced sneezing, itchy nose, runny nose, or nasal congestion in the past 12 months? (You may select multiple options)**

- January - July
- February - August
- March - September
- April - October
- May - November
- June - December

**31) In the past 12 months, have you experienced nose or eye symptoms, without having a cold, when exposed to any of the following?**

- No, I have not had symptoms from any of these
- Pets
- Birch pollen
- Grass pollen
- Mugwort pollen
- Dust mites
- Mold
- During the period from August 1st to November 1st
- Hiking in the forest between August 1st and November 1st
- Other

**32) Have you had difficulty sleeping due to nasal symptoms in the past 12 months?**

- No
- Yes

**33) In the past 12 months, have you taken any medication for allergic rhinitis/hay fever?**

- No, go to question 35
- Yes

**34) What medications for allergic rhinitis/hay fever have you used in the past 12 months?**

- Eye drops: Livostin, Zaditen, Opatanol, Emadine, Spersallerg, Lomudal, or Lecrolyn
- Nasal spray: Rhinox, Otrivin, Dexyl, Zymelin, Otrivin comp, or Zycomb
  - Antihistamine nasal spray: Livostin
- Cortisone nasal spray: Avamys, Nasonex, Flutide nasal, Budesonide, Rhinocort, Fluticasone, Mometasone, or Nasacort
  - Cortisone + Antihistamine nasal spray: Dymista
- Antihistamine tablets: Zyrtec, Cetirizine, Aerius, Telfast, Xysal, Loratadine, Clarityn, Ebastine, or Kestine
  - Cortisone tablets: Prednisolone or Betapred
  - Cortisone injection(s)

**35) Have you had nasal congestion in the past 12 months?**

- No
- Less than 10 days
- 10 days – 12 weeks
- 12 weeks or more

**36) Have you had mucus at the back of your throat in the past 12 months?**

- No
- Less than 10 days
- 10 days – 12 weeks
- 12 weeks or more

**37) Have you had reduced sense of smell in the past 12 months?**

- No
- Less than 10 days
- 10 days – 12 weeks
- 12 weeks or more

**38) Have you experienced pain or pressure around your forehead, nose, or eyes in the past 12 months?**

- No
- Less than 10 days
- 10 days – 12 weeks
- More than 12 weeks

**39) During which period in the past 12 months have you had symptoms of mucus at the back of your throat, reduced sense of smell, or pain/pressure around your forehead, nose, or eyes?** (You can select multiple options)

- January - July
- February - August
- March - September
- April - October
- May - November
- June - December

**40) If you have nasal or eye symptoms, have they:**

- Prevented you from doing schoolwork/work?
- Prevented you from participating in leisure activities?
- Prevented you from being outdoors?

**Family and doctor-diagnosed atopic diseases**

**41) Does anyone in your family (parents, siblings, children, or grandchildren) currently have or have had any of the following?**

- No, none of them
- Asthma
- Eczema
- Hay fever/respiratory allergies
- Food allergies

**42) Have you ever been diagnosed by a doctor with any of these conditions?**

- No, none of them
- Asthma
- Eczema
- Hay fever/respiratory allergies
- Food allergies

**43) Did you have any of these conditions as a child or adolescent?**

- No, none of them
- Asthma
- Eczema
- Hay fever/respiratory allergies
- Food allergies

**Supplementary 2: Outdoor pollen and spore dispersal**

**Monitoring of pollen and spores**

Since 1980, the Norwegian National Pollen Forecast, operated by the Norwegian University of Science and Technology (NTNU), has conducted daily monitoring of airborne pollen and fungal spores across Norway. Measurements are currently performed at 12 monitoring stations, including Bodø, Tromsø, and Kirkenes in Northern Norway. Air samples are collected using volumetric Burkard spore traps and analysed according to standard aerobiological monitoring procedures described in the national monitoring reports (1). Pollen and spore concentrations are expressed as pollen grains/m³ and spores/m³ of air, respectively, and particle identification and counting are performed using light microscopy. While a substantial proportion of particles typically originate from regional vegetation sources, long-distance transport may also contribute to observed concentrations depending on meteorological conditions (1).

Birch rust (BR) spores have been included in the Norwegian monitoring program since 2004 as part of the routine surveillance of airborne fungal spores. BR spores are morphologically distinctive and can be readily identified in the collected samples. However, they have not previously been considered a potential trigger of seasonal allergic symptoms (1,2). Monitoring practices vary across European pollen and spore networks, and the fungal taxa included in routine analyses differ between regions, although genera such as *Alternaria* and *Cladosporium* are commonly monitored. To our knowledge, systematic monitoring of BR spores has rarely been reported elsewhere in Europe (3,4).

Using daily BR registrations from monitoring stations in Bodø, Tromsø and Kirkenes, seasonal spore counts were compared with the average symptom severity reported by patients associated with each study sites. This approach allows for a more detailed analysis of the potential correlation between BR spore presence and seasonal airway allergy symptoms. However, due to the absence of monitoring stations in Alta and Mo i Rana, spore data were unavailable for these locations, precluding a comparison of symptoms and BR spore distribution in these areas.

**References**

1.	Ramfjord H, Brobakk TE. Registrering av pollen og sporer. Astma og Allergiforbundet, NTNU, Institutt for biologi, Helsedirektoratet; 2020–2023. [https://www.naaf.no/allergi/pollenallergi/pollenrapporter](https://eur01.safelinks.protection.outlook.com/?url=https%3A%2F%2Fwww.naaf.no%2Fallergi%2Fpollenallergi%2Fpollenrapporter&data=05%7C02%7C%7C3448865c4a334312c46f08de72ad10e4%7C677181082eb7474ca1d741655d4b1e61%7C0%7C0%7C639074284786950903%7CUnknown%7CTWFpbGZsb3d8eyJFbXB0eU1hcGkiOnRydWUsIlYiOiIwLjAuMDAwMCIsIlAiOiJXaW4zMiIsIkFOIjoiTWFpbCIsIldUIjoyfQ%3D%3D%7C0%7C%7C%7C&sdata=9qF%2FOp%2BOtzNNtZhmcqv0HmE3ODK9qxpi63EPMm4Oo3c%3D&reserved=0)

2.	Frisk CA, Brobakk TE, Ramfjord H. Allergenic pollen seasons and regional pollen calendars for Norway. Aerobiologia. 2024.

3.	Anees-Hill S, Douglas P, Pashley CH, Hansell A, Marczylo EL. A systematic review of outdoor airborne fungal spore seasonality across Europe and the implications for health. Sci Total Environ. 2022;818:151716.

4.	Buters JTM, Antunes C, Galveias A, Bergmann KC, Thibaudon M, Galán C, et al. Pollen and spore monitoring in the world. Clin Transl Allergy. 2018;8:9.

**Supplementary 3: Definitions Table 1**

**Current asthma:** At least two of the following 3 criteria: 1) self-reported doctor-diagnosed asthma ever, 2) any indicative symptom in the last 12 months (wheezing, shortness of breath, dry cough at night), and 3) use of asthma medication in the past 12 months (1).

**Current rhinitis:** At least two of the following 3 criteria: 1) self-reported doctor-diagnosed allergic rhinitis ever, 2) symptoms of sneezing, a runny or blocked nose, and 3) itchy, red and watery eyes after exposure to furred pets, pollen or house dust mite (2).

**Current eczema:** At least two of the following 3 criteria: 1) self-reported doctor-diagnosed eczema ever, 2) dry skin, itchy rashes for two weeks or more in the past 12 months, and 3) self-reported eczema combined with use of topical corticosteroids in the past 12 months (2).

**Anti-allergic treatment**: Oral, nasal, and/or conjunctival antihistamines and/or steroids, plus steroid injections.

**Anti-asthmatic treatment:** Mono- or combination therapy with short- or long-acting beta-2 agonists, muscarinic receptor antagonists, and/or inhaled corticosteroids, and/or leukotriene antagonists, and/or oral steroids.

**In BR season:** At least 6 days with anti-allergic or anti-asthmatic treatment.

**Outside BR season:** At least 2 days with anti-allergic or anti-asthmatic treatment.

**Proximity to birch forest:** Defined as having been near or having spent time in an area perceived by the participant as birch forest.

**References**

1.	Gough H, Grabenhenrich L, Reich A, Eckers N, Nitsche O, Schramm D, et al. Allergic multimorbidity of asthma, rhinitis and eczema over 20 years in the German birth cohort MAS. Pediatr Allergy Immunol. 2015;26(5):431–7.

2.	Ballardini N, Bergström A, Wahlgren C-F, van Hage M, Hallner E, Kull I, et al. IgE antibodies in relation to prevalence and multimorbidity of eczema, asthma, and rhinitis from birth to adolescence. Allergy. 2016;71(3):342–9.

**Supplementary 4: Symptom Registration Data, PNIF, and Spirometry Results.**

|  | | Patients (N=160) | Controls (N=94) | P-values |
| --- | --- | --- | --- | --- |
| **Allergic symptoms** | | | | |
| Total Symptoms (mean VAS)^1^ | |  |  |  |
|  | In BR season^5^ | 2.86 (SD 1.66) | 0.10 (SD 0.30) | <0.001^†^ |
|  | Outside BR season^6^ | 1.12 (SD 1.46)* | 0.06 (SD 0.24) | <0.001^†^ |
| Nasal Symptoms (mean VAS)^2^ | |  |  |  |
|  | In BR season^5^ | 2.74 (SD 1.71) | 0.19 (SD 0.34) | <0.001^†^ |
|  | Outside BR season^6^ | 1.28 (SD 1.54)* | 0.24 (SD 0.58) | <0.001^†^ |
| Eye Symptoms (mean VAS)^3^ | |  |  |  |
|  | In BR season^5^ | 1.91 (SD 1.71) | 0.03 (SD 0.10) | <0.001^†^ |
|  | Outside BR season^6^ | 0.75 (SD 1.29)* | 0.05 (SD 0.21) | <0.001^†^ |
| Asthma Symptoms (mean VAS)^4^ | |  |  |  |
|  | In BR season^5^ | 1.63 (SD 1.83) | 0.08 (SD 0.33) | <0.001^†^ |
|  | Outside BR season^6^ | 0.66 (SD 1.33)* | 0.12 (SD 0.48) | <0.001^†^ |
| **Medical treatment** | | | | |
| Mean duration of anti-allergic treatment (days)^7^ | |  |  |  |
|  | In BR season^5^ | 19.5 (SD 16.5) | 0.2 (SD 1.7) | <0.001^†^ |
|  | Outside BR season^6^ | 5.6 (SD 7.5)* | 0.2 (SD 1.8) | <0.001^†^ |
| Mean duration of anti-asthmatic treatment (days)^8^ | |  |  |  |
|  | In BR season^5^ | 10.5 (SD 16.3) | 0 | <0.001^†^ |
|  | Outside BR season^6^ | 4.0 (SD 7.1)* | 0 | <0.001^†^ |
| **Airway Function** | | | | |
| PNIF (L/min, mean values)^9^ | |  |  |  |
|  | In BR season | 115.1 (SD 35.7) | 127.2 (SD 35.4) | 0.009^†^ |
|  | Outside BR season | 120.3 (SD 35.8) | 126.4 (SD 34.0) | 0.18^†^ |
| FEV1 (L/min, mean values)^9^ | |  |  |  |
|  | In BR season | 3.03 (SD 0.79) | 3.25 (0.67) | 0.03^†^ |
|  | Outside BR season | 3.03 (SD 0.78) | 3.22 (SD 0.65) | 0.05^†^ |
| FVC (L/min, mean values)^9^ | |  |  |  |
|  | In BR season | 3.89 (SD 0.98) | 4.12 (SD 0.84) | 0.05^†^ |
|  | Outside BR season | 3.89 (SD 0.96) | 4.11 (SD 0.85) | 0.08^†^ |
| PEF (L/min, mean values)^9^ | |  |  |  |
|  | In BR season | 474.6 (SD 106.1) | 491.3 (SD 92.1) | 0.21^†^ |
|  | Outside BR season | 482.3 (SD 106.2) | 501.3 (SD 91.9) | 0.15^†^ |

**Abbreviations:** BR, birch rust; SD, standard deviation; VAS, visual analogue scale.
**Statistical tests:** † Student`s *t* test.

^1^: Grading of overall allergic symptoms.

^2^: Grading of rhinitis symptoms: sneezing, itching, runny nose, nasal congestion, forehead pressure/pain, and throat mucus.

^3^: Grading of conjunctivitis symptoms: itching, redness, and tearing.

^4^: Grading of asthma symptoms: chest tightness, coughing, wheezing, and shortness of breath.

^5^: Total registration period of 47 days in the season of BR spore dispersal, from August 15 September 30, 2022.

^6^: Total registration period of 19 days outside the season of BR spore dispersal, from January 15 to February 3, 2023.

^7^: Oral, nasal, and/or conjunctival antihistamines and/or steroids, plus steroid injections.

^8^: Mono- or combination therapy with short- or long-acting beta-2 agonists, muscarinic receptor antagonists, and/or inhaled corticosteroids, and/or leukotriene antagonists, and/or oral steroids.

^9^: One measurement per participants each season.

*N=159 due to missing symptom registration outside the BR season for one patient.

***Supplementary 5: Comparison of Symptom Seasonality between Patients* and Controls: Variations in Symptoms Across Seasons^1^.***

|  | ***Model 0*** | ***Model 1*** |
| --- | --- | --- |
| ***Total Symptoms (VAS)^2^*** | 1.70, p<0.001 | 1.13, p<0.001 |
| ***Nasal Symptoms (VAS)^2^*** | 1.50, p<0.001 | 0.99, p=0.001 |
| ***Eye Symptoms (VAS)^2^*** | 1.18, p<0.001 | 0.75, p=0.001 |
| ***Asthma Symptoms (VAS)^2^*** | 0.99, p<0.001 | 0.44, p=0.10 |

**Abbreviations:** BR, birch rust; SD, standard deviation; VAS, visual analogue scale; PNIF, peak nasal inspiratory flow; L, Liter; L/min, Liter/minute.

**Statistical analysis:** Linear regression models: Model 0, unadjusted; Model 1, adjusted for proximity to birch forest, anti-allergic and anti-asthmatic treatment, rhinitis in the last 12 months, asthma in the last 12 months, pollen allergy, perennial allergy, family history of atopy, and ever atopic disease.

^1^Patients (mean symptom VAS score in BR season – mean symptom VAS score outside BR season) – Controls (mean symptom VAS score in BR season – mean symptom VAS score outside BR season).

^2^For background data, see Supplementary 6.

*N=159 due to missing symptom registration outside the BR season for one patient.

***Supplementary 6: Seasonal Variations in allergic symptoms and airway obstruction, stratified by study groups***

| ***Seasonality^1^*** | ***Patients**** | | | ***Controls*** | | |
| --- | --- | --- | --- | --- | --- | --- |
|  | ***Model 0*** | ***Model 1*** | ***Model 2*** | ***Model 0*** | ***Model 1*** | ***Model 2*** |
| ***Total Symptoms (VAS)*** | 1.74, p<0.001 | 1.15, p<0.001 | 0.98, p<0.001 | 0.04, p=0.34 | 0.03, p=0.47 | 0.03, p=0.41 |
| ***Nasal Symptoms (VAS)*** | 1.46, p<0.001 | 0.89, p<0.001 | 0.78, p<0.001 | -0.05, p=0.48 | -0.08, p=0.30 | -0.07, p=0.34 |
| ***Eye Symptoms (VAS)*** | 1.17, p<0.001 | 0.68, p<0.001 | 0.55, p=0.001 | -0.03, p=0.29 | -0.02, p=0.38 | -0.02, p=0.41 |
| ***Asthma Symptoms (VAS)*** | 0.97, p<0.001 | 0.40, p=0.05 | 0.35, p=0.04 | -0.04, p=0.50 | -0.06, p=0.37 | -0.04, p=0.46 |
| ***PNIF (L/min)*** | -5.25, p=0.01 | -5.72, p=0.19 |  | 0.85, p=0.76 | 2.15, p=0.69 |  |
| ***FEV1 (L)*** | 0.004, p=0.72 | 0.009, p=0.93 |  | 0,028, p<0.001 | 0.053, p=0.60 |  |
| ***FVC (L)*** | -0.009, p=0.53 | -0.021, p=0.86 |  | 0.019, p=0.18 | 0.023, p=0.86 |  |

**Abbreviations:** VAS, visual analogue scale; PNIF, peak nasal inspiratory flow; L, Liter; L/min, Liter/minute.

**Statistical analysis:** Linear regression models: Model 0, unadjusted; Model 1, adjusted for proximity to birch forest; Model 2, adjusted for proximity to birch forest, anti-allergic and anti-asthmatic treatment, rhinitis in the last 12 months, asthma in the last 12 months, pollen allergy, perennial allergy, family history of atopy, and ever atopic disease.

^1^Seasonality = mean VAS/PNIF/FEV1/FVC score in BR season – mean symptom VAS/PNIF/FEV1/FVC score outside BR season.

*N=159 due to missing symptom registration outside the BR season for one patient.
